# Preparation of a camptothecin analog FLQY2 self-micelle solid dispersion with improved solubility and bioavailability

**DOI:** 10.1186/s12951-022-01596-2

**Published:** 2022-09-05

**Authors:** Yi Wang, Wenchao Wang, Endian Yu, Wenya Zhuang, Xuanrong Sun, Hong Wang, Qingyong Li

**Affiliations:** College of Pharmaceutical Sciences, Zhejiang University of Technology, No. 1 Gongda Road, Hangzhou, 313000 People’s Republic of China

**Keywords:** Camptothecin, Soluplus^®^, Solid dispersion, Micelle, Solubility, Pharmacokinetics, In vivo antitumor activity

## Abstract

**Background:**

7-p-trifluoromethylphenyl-FL118 (FLQY2) is a camptothecin analog with excellent antitumor efficacy against various solid tumors. However, its poor solubility and low bioavailability limited the development of the drug. Polyvinyl caprolactam-polyvinyl acetate-polyethylene glycol graft copolymer (Soluplus^®^), an emerging carrier for preparing solid dispersion (SD), encapsulated FLQY2 to circumvent the above limitations.

**Results:**

In this project, FLQY2-SD was prepared by solvent evaporation method and self-assembled into micelles in aqueous solutions owing to the amphiphilic nature of Soluplus^®^. The physicochemical characterizations demonstrated that FLQY2 existed in a homogeneous amorphous form in SD and was rapidly dissolved. The micelles did not affect cytotoxicity or cellular uptake of FLQY2 in vitro, and the oral bioavailability was increased by 12.3-fold compared to the FLQY2 cyclodextrin suspension. The pharmacokinetics of FLQY2-SD showed rapid absorption, accumulation in the intestine, and slow elimination via fecal. Metabolite identification studies showed 14 novel metabolites were identified, including 12 phase I metabolites (M1–M12) and 2 phase II metabolites (M13–M14), of which M2 (oxidation after decarboxylation) and M7 (dioxolane ring cleavage) were the primary metabolites in the positive mode and negative mode, respectively. The tumor growth inhibition rate (TGI, 81.1%) of FLQY2-SD (1.5 mpk, p.o./QW) in tumor-bearing mice after oral administration was higher than that of albumin-bound Paclitaxel (15 mpk, i.v./Q4D) and Irinotecan hydrochloride (100 mpk, i.p./QW).

**Conclusions:**

The successful preparation, pharmacokinetics, and pharmacodynamics studies of FLQY2-SD showed that the solubility and bioavailability of FLQY2 were improved, which facilitated the further druggability development of FLQY2.

**Graphical Abstract:**

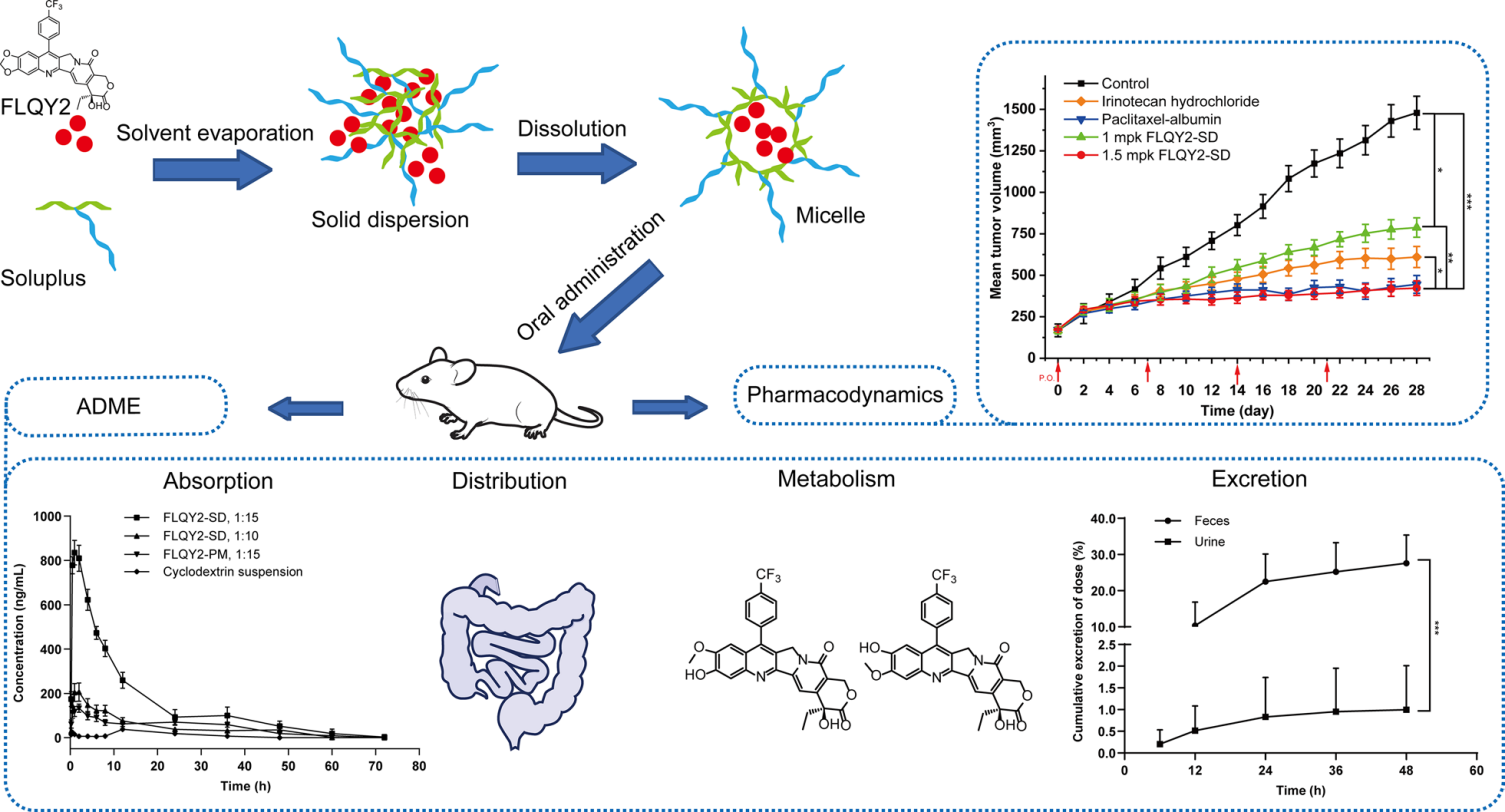

**Supplementary Information:**

The online version contains supplementary material available at 10.1186/s12951-022-01596-2.

## Introduction

10,11-methylenedioxy-20(S)-camptothecin synthesized by Wall’s group exhibited greater Topoisomerase I inhibition potency and in vivo antitumor activity than 20(S)-CPT, but its high toxicity hindered further studies [1]. In 2012, 10,11-methylenedioxy-20(S)-CPT was rediscovered and designated FL118 as a molecule targeting survivin (a protein in the inhibitor of apoptosis family) via high-throughput screening [2]. Subsequent studies have shown that FL118 possessed superior antitumor activity, including colon cancer [2–5], head & neck cancer [2, 3], and pancreatic cancer [6]. Moreover, the mechanism of FL118 was found to inhibit survivin, Mcl-1, XIAP, and cIAP2 by genetic silencing or overexpression [2, 4]. Compared to FDA-approved CPT analogs (e.g., irinotecan and topotecan), FL118 could reverse multidrug resistance by bypassing the efflux pump proteins ABCG2/BCRP and ABCB1/P-gp [7–9].

Some new derivatives were developed using FL118 as the platform, such as introducing lipophilic substituents at the C-7 position [10]. Based on the structure–activity relationship of FL118, FLQY2 (7-p-trifluoromethylphenyl-FL118) was synthesized by our laboratory. It has been proved that FLQY2 possessed better antitumor activity and permeability than FL118 on HCT 116 and Hep G2 cell lines with nM level IC_50_ [11]. Further transport study has revealed that the transport absorption of FLQY2 mainly relied on passive diffusion while the efflux of P-gp was slight (efflux ratio  < 2) [12]. Despite possessing the above pharmacological activities, FLQY2 was a water-insoluble molecule, which could not be detected in water by HPLC. And earlier studies showed less improvement in the bioavailability of FLQY2 cyclodextrin suspensions [13], so a new formulation of FLQY2 was necessary. Besides, candidate metabolism is highly correlated with drug efficacy, so the research on FLQY2 metabolism is also important [14].

To overcome the drawbacks of CPT analogs, many drug delivery systems strategies, such as liposomes [15, 16], micelles [17, 18], and SD [19, 20], have been developed. Among these strategies, SD technology was one of the most promising and widely accepted methods over the decades, which could improve drug release while possessing the advantages of particle size reduction, superficial expansion, crystallization inhibition, and enhanced wettability and porosity [21]. Moreover, the drug in amorphous SD is molecularly dispersed in a high-energy form, and increases the apparent solubility and overcoming a solubility-permeability [22, 23]. The third generation of SD was prepared using surface-active agents like Poloxamer, Gelucire 44/14, and Soluplus^®^, designed to improve the drug wettability and avoid drug recrystallization [24, 25]. An amphiphilic polymer Soluplus^®^ was developed by BASF for melt extrusion [26]. In recent years, 9-NC-SD using Soluplus^®^ was prepared by freeze-drying method [19] and CPT-SD was prepared by solvent evaporation method [27]. Soluplus^®^ can quickly form polymeric micelles that encapsulate the drug in water, and achieve superior solubilization and thermodynamic stability. Besides, the micelles are more resistant to dilution effects due to the low critical micelle concentration (CMC) of 7.6 mg/L [28–30]. Moreover, polymer micelles with a size of less than 200 nm decrease the clearance by the non-selective reticuloendothelial system and improve the penetration of passive targeting at solid tumor sites [31].

Briefly, the main objective of this work was to try a novel formulation in the form of SD for the water-insoluble FLQY2 to improve its solubility and bioavailability. The carriers were screened, the preparation process was optimized, and the characterization, the biological behavior of the optimal FLQY2-SD micelles in an aqueous solution was investigated, including cellular uptake, cytotoxic activity, pharmacokinetics, tissue distribution, excretion, metabolic profiles, and in vivo efficacy.

## Materials and methods

### Materials

FLQY2 was synthesized by our laboratory (purity  > 98%) [32]. Soluplus^®^, Poloxamer 407, PVP Va64, and HS 15 were purchased from BASF Co., Ltd (Shanghai, China). PEG4000, PEG6000, and Irinotecan hydrochloride were obtained from Aladdin Bio-chem Technology Co., Ltd (Shanghai, China). Paclitaxel Injection (Albumin-Bound) was purchased from Kelun Pharmaceutical Co., Ltd (Yueyang, China). Human colon cancer cells (HCT 116) and human pancreatic cancer cells (MIA PaCa-2) were acquired from the National Collection of Authenticated Cell Cultures (Shanghai, China). McCoy’s 5A medium and DMEM medium were obtained from SIGMA (Shanghai, China). FBS was purchased from Gibco (Shanghai, China). Penicillin–streptomycin solution (100 ×) was obtained from Solarbio Co., Ltd (Beijing, China). Acetonitrile and ultrapure water for HPLC-grade were purchased from Merck (Darmstadt, Germany). HPLC-grade formic acid and ammonium acetate were purchased from CNW (Shanghai, China). All the other chemicals were of analytical grade and commercially available.

### Preparation of FLQY2-SD

#### Screening of carriers

FLQY2 and six different carriers (Soluplus^®^, Poloxamer 407, PVP Va64, HS 15, PEG4000, and PEG6000) were weighed at the ratio of 1:20 and then dissolved respectively in 4 mL methylene chloride and stirred for 30 min at 37 °C. The solution was evaporated to obtain the solid matter and dried overnight under a vacuum at 50 °C. The solid matter was smashed and the powder was sieved through an 80# sieve. The mixture consisting of 3 mL of pure water and the six kinds of powder (including 1 mg FLQY2) was shaken for 4 h at 37 °C. The solution was filtered through 0.22 μm filters and diluted by mobile phase (methanol (A)/water containing 0.01% formic acid (B), 8:2), then the amount of the dissolved FLQY2 in six different carriers was determined by HPLC.

#### Inhibition of crystallization

Inhibition of crystallization was studied using the solvent-shift method [33]. 3 mL FLQY2 DMSO solution (1 mg/mL) was mixed respectively with six different 30 mL aqueous solution including six carriers (2 mg/mL) and stirred at 120 r/min under 37 °C. 0.5 mL solution was collected at 0.0833, 0.5, 1, 2, 4, 6, 8 h, filtered through 0.22 μm filters, and determined the dissolved FLQY2 by HPLC.

#### Measurement of saturation solubility

FLQY2 and Soluplus^®^ were weighed at the ratios of 1:5, 1:10, 1:12, 1:15, 1:18, 1:20, 1:25, and 1:30 (w/w). And the SD powder was prepared according to the “screening of carriers” section. The excessive powder was added to 10 mL pure water and shaken for 12 h at 37 °C at 120 r/min. The supernatant was filtered, diluted, and determined the dissolved FLQY2 by HPLC.

#### Preparation of the FLQY2 different formulations

FLQY2-SD powder (SD) was prepared according to the “screening of carriers” section (FLQY2: Soluplus^®^, 1:15). FLQY2 and Soluplus^®^ were pulverized and mixed at a weight ratio of 1:15 and sieved through sieve # 80 to prepare a physical mixture (PM). FLQY2 and Soluplus^®^ were mixed at a weight ratio of 1:15 to prepare a direct mixture (DM). The solution α was prepared by mixing 6.27 g of PEG400 and 110.46 mL of saline solution. The solution β was prepared by mixing 10.20 g of β-cyclodextrin and 25.00 mL of DMSO. 2 mg of FLQY2, 1860 μL of the solution α, and 140 μL of the solution β were ultrasonically mixed to prepare the FLQY2 cyclodextrin suspension using the literature [13].

### Characterization

The particle size and polydispersity index (PDI) of FLQY2-SD micelle solution were determined by a Zetasizer Nano ZS90 (Malvern Instruments, UK). FLQY2, Soluplus^®^, PM, and FLQY2-SD powder were performed by x-ray diffraction (XRD) (X’Pert Pro, PANalytical Co., Holland). The measurement conditions were as follows: Cu Kα rays, 0.1541 nm; voltage, 40 kV; current, 40 mA; scan range 2θ, 3–40°; and step size, 0.0167°. FLQY2, Soluplus^®^, PM, and FLQY2-SD powder were analyzed using differential scanning calorimetry (DSC) (Discovery, Jing Yi Chemical Materials Co., Ltd, China). Each sample was weighed at 3 mg and sealed in the crucible aluminum tray. Detection conditions were listed below: scanning range, 50 ~ 350 °C; temperature rise rate, 10 °C/min; protective gas, nitrogen; the flow rate, 50 mL/min. FLQY2, Soluplus^®^, PM, and FLQY2-SD powder were analyzed using infrared spectral analysis (IR) (Nicolet 6700, Thermo Fisher Scientific, USA). To obtain the corresponding IR spectra, each sample powder was appropriately mixed with dried KBr, pressed into tablets, and scanned in the wavelength range of 4000 ~ 400/cm.

#### Release experiment in vitro

The FLQY2-SD powder was dissolved completely in the tested media for 30 min to form micelles solution by HPLC detection. The release behavior of FLQY2-SD micelles solution in vitro was investigated by dynamic dialysis. Three initial concentrations of FLQY2-SD micelles solution (including FLQY2 12.5, 50, 125 μg/mL respectively), two weight ratios between FLQY2 and Soluplus^®^ (1: 15, 1: 20), and three pH of media (4.1, 5.5, 6.75) were tested. In short, a specific concentration of FLQY2-SD micelles solution mixed with 40 mL dialysis solution containing 10% Tween80 was added into a dialysis bag (MWCO = 3500 Da) at a rotation speed of 120 r/min and 37 °C. 5 mL of the sample was taken at 0.5, 1, 2, 4, 6, 8, 12, 24, 36, 48, 60, 72 h and the equal volume of fresh medium was replenished. The cumulative release Er (%) of FLQY2 is calculated as below.

$${E}_{r}=\frac{{V}_{e}{\sum }_{1}^{n-1}{C}_{i}+{V}_{0}{C}_{n}}{{m}_{comp}}$$

where V_e_ is the displacement volume of dialysate (mL); V_0_ is the total volume of dialysate (mL); C_i_ is the concentration of FLQY2 in dialysate at the sample (μg/mL), and m_comp_ is full content of FLQY2 in micelles (μg/mL).

### Biological evaluation

The sample preparation: FLQY2 was dissolved in DMSO as a stock solution, and the stock solution was diluted by DMEM medium as a working solution. FLQY2-SD/ FLQY2-PM powder was dissolved in water, then the FLQY2-SD/FLQY2-PM solution was diluted by DMEM medium.

#### Cell culture and in vitro anticancer activity

The cells were cultured in McCoy’s 5A medium for HCT 116 or DMEM medium for MIA PaCa-2 cells. All mediums contained 10% FBS and 1% penicillin–streptomycin solution. The cytotoxicity of FLQY2/FLQY2-SD was measured against these cells using an MTT assay. Briefly, 1 × 10^4^ cells per well were seeded into 96 well plates for 24 h. Different concentrations of FLQY2 /FLQY2-SD solution and blank fresh medium were added to the plates and then incubated for 72 h. After adding MTT solution (5 mg/mL) for 4 h, 150 μL of DMSO was added to dissolve the formazan crystals. Absorbance at 570 nm with the control wavelength of 630 nm was used in infinite M200 pro (Tecan, Switzerland). Cell viability was expressed as a percentage relative to the blank control.

#### Morphology study

HCT 116 and MIA PaCa-2 cells were seeded in 35 mm Confocal Dishes (BeyoGold, China) at a density of 1 × 10^4^/per dish and cultured overnight. FLQY2-SD micelles solution was added to the dishes and then incubated for 72 h. Morphological cytotoxicity results were obtained before and after administration by a holographic microscope optical tomography (Nanolive 3D Cell Explorer, Switzerland).

#### Cellular uptake assay

HCT 116 and MIA PaCa-2 cells were seeded into 12 well plates at a density of 1 × 10^5^/per well for 48 h. FLQY2/FLQY2-SD /FLQY2-PM (1 µmol/L) were added to the plates and then incubated for 15, 30, 45, 60, 90, and 120 min at a specific temperature. The cells were rinsed, collected, and lysed with the medium removed after three freeze–thaw cycles. The concentration of protein was determined by the Coomassie Brilliant Blue method, while the concentration of FLQY2 was determined by HPLC.

#### Pharmacokinetics study

30 female Sprague–Dawley rats were randomly and equally divided into 5 groups (n = 6). The blood samples were collected from the four groups at 0.25, 0.5, 1, 2, 4, 6, 8, 12, 24, 36, 48, 60, and 72 h after being orally administered FLQY2-SD micelles solution/FLQY2-PM solution/FLQY2 cyclodextrin suspension (8 mg/kg FLQY2), with one group serving as a blank control. 200 µL of samples were vortexed with 580 µL of ice-cold precipitant (methanol: acetonitrile = 2: 1, containing 0.1% acetic acid), and 20 µL of FL77-32 served as an internal standard (IS). After centrifugation at 10,000 rpm for 10 min, the supernatant was filtered, diluted, and determined the dissolved FLQY2 by HPLC. Pharmacokinetic parameters were calculated by PK solver software.

#### Tissue distribution and excretion study

Twelve male and twelve female Sprague–Dawley rats were randomly and equally divided into six groups (n = 4). Five groups of rats were sacrificed at 0.5, 1.5, 8, 24, and 48 h after being orally administered with FLQY2-SD micelles solution (4 mg/kg FLQY2), with one group serving as a blank control. The heart, stomach, liver, intestine, spleen, pancreas, lung, muscle, kidney, and brain tissues were separated, rinsed, and weighed before homogenate. The rest of the operations were the same as in the “Pharmacokinetics study” section.

Two male and two female Sprague–Dawley rats were placed in the metabolic cages, and their blank fecal and urine samples were collected. The Sprague–Dawley rats were sacrificed at 48 h after being orally administered with FLQY2-SD micelles solution (4 mg/kg FLQY2). The urine samples were collected at 0–6, 6–12, 12–24, 24–36, and 36–48 h. The rest of the operations were the same as in the “Pharmacokinetics study” section.

For feces, the samples were collected at 0–12, 12–24, 24–36, and 36–48 h and freeze-dried. 1 mL methanol was added to 100 mg feces, ultrasonicated for 10 min, and centrifuged at 10,000 rpm for 10 min. 50 µL of supernatant with the 730 µL of precipitant and 20 µL of IS were vortexed for 3 min, the rest of the operations were the same as in the “Pharmacokinetics study” section.

#### Identification of metabolites

The plasma, urine, and feces samples were processed as described above without IS, and the remaining volumes were made up of the precipitant. Chromatographic analysis was performed on an UltiMate 30 (Thermo Fisher Scientific, San Jose, CA, USA). Separation was carried out on an ACQUITY UPLC HSS T3 (1.8 μm, 2.1 × 100 mm) at 40 °C, 2 µL injection volume. In the positive ion mode, the mobile phase consisted of water containing 0.1% formic acid (A) and acetonitrile containing 0.1% formic acid (B). The gradient elution was performed with 5% B for 1.5 min, increasing to 10% B at 2.5 min, then ramping to 40% B at 14 min, rising to 95% B at 22 min, and remaining at 95% B until 25 min, and finally, the column was reconditioned with 5% B for 5 min. The mobile phase consisted of water containing 2 mM ammonium acetate (A) and acetonitrile (B) in the negative ion mode. The gradient elution was the same as in the positive ion mode.

Mass spectrometric analysis was acquired by Peakview 2.2 and MetabolitePilot 2.0.4 software using a quadrupole time-of-flight mass spectrometry (TripleTOF^™^ 5600, AB Sciex Corporation., Foster City, CA) with MS scans and information dependent acquisition (IDA) MS/MS scans. The mass spectrometer was operated in both positive and negative ion mode with electrospray ionization (ESI) source at 650 ℃. The detector conditions of the MS were listed below: ion source gas1 was 60 psi; ion source gas2 was 60 psi; curtain gas was 35 psi; ion spray voltage floating was 5000 V in the positive ion mode or −4000 V in the negative ion mode; collision energy was 30 eV. TOF/MS range was conducted in *m/z* 50–1200.

#### Anticancer activity in vivo

0.1 mL suspension of HT-29 cells were inoculated in female BALB/c mice at a density of 2 × 10^7^/mL. After the tumors grew to 100–200 mm^3^ (the day recorded as D_0_), the mice were randomly grouped and administered with Irinotecan hydrochloride (100 mpk, i.p./QW) (n = 6), Paclitaxel-albumin (15 mpk, i.v./Q4D) (n = 6), FLQY2-SD (1.5 mpk, p.o./QW) (n = 6), FLQY2-SD (1 mpk, p.o./QW) (n = 6), and saline as a blank control (n = 12). The tumor volume and body weight of the mice were recorded every 2 days. On day 28, the mice were sacrificed, and the tumors were surgically removed and weighed. Some parameters were calculated as follows.

tumor volume (TV): $TV=\frac{1}{2}\times a{b}^{2}$

relative tumor volume (RTV): $RTV=\frac{{V}_{t}}{{V}_{0}}$

tumor growth inhibition (TGI): $TGI=(1-\frac{{V}_{{T}_{28}}-{V}_{{T}_{0}}}{{V}_{{C}_{28}}-{V}_{{C}_{0}}})\times 100\%$

Where a and b are the length and width of the tumors; V_0_ and V_t_ are the tumor volume measured at D_0_ and at each measurement time D_t_; V_T28_ and V_T0_ are the mean tumor volume in the treatment group at D_28_ and D_0_; V_C28_ and V_C0_ are the mean tumor volume in the negative control group at D_28_ and D_0_.

#### HPLC methods

All quantification analyses were measured by HPLC. The instrument and conditions were as follows: liquid chromatography pump (LC-20AT, Shimadzu, Japan), UV detector (SPD-20A, Shimadzu, Japan), column oven (CTO-10AS, Shimadzu, Japan) manual sample injector valve (9725i, Rheodyne, USA), SinoChrom ODS-BP column (250 mm × 4.6 mm × 5 μm, Elite Analytical Instruments Co., Ltd, China), column temperature of 40 °C, the flow rate of 1 mL/min, the wavelength of 386 nm. The mobile phase consisted of methanol (A)/water containing 0.01% formic acid (B) with isocratic elution, varying the volume ratio according to the sample (V_A_ = 80–74%).

### Statistical analysis

The data were expressed as the mean value ± standard deviation. Statistical differences were assessed using Student’s t-test.

## Results and discussion

### Screening of carriers

**Fig. 1:**
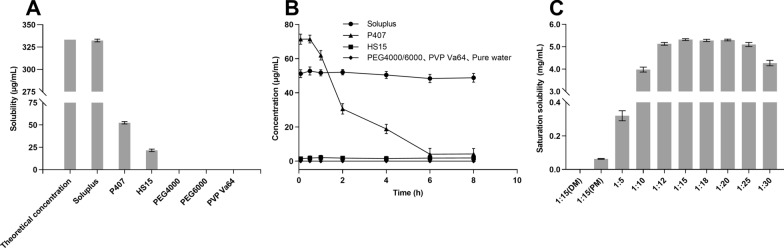
**A** Solubility **B** inhibition of crystallization of FLQY2 encapsulated with different carriers. **C** Saturation solubility of FLQY2 at different drug-Soluplus^**®**^ ratios

The solubility of drugs can be improved by carriers with excellent affinity and miscibility [34]. In addition, the ease of drug crystallization is governed by drug-polymer carrier interactions [25]. Therefore, the selection of a suitable polymer carrier is crucial. In this part, the solubility and crystallization inhibition were measured to screen the carriers for FLQY2. As shown in Fig. 1A, the solubilities of FLQY2 in PEG4000, PEG6000, and PVP Va64 were less than 0.5 μg/mL. The solubilities of FLQY2 in HS 15 and P407 reached 21.7 ± 1.4 μg/mL and 52.5 ± 1.3 μg/mL, respectively. The solubilities of FLQY2 in Soluplus^®^ were remarkably 332.3 ± 1.6 µg/mL, indicating that the tested FLQY2 was almost completely dissolved in the Soluplus (Fig. 1A). In the crystallization inhibition assay, a FLQY2 DMSO solution (1 mg/mL) was added to six kinds of an aqueous solution containing respectively the six different carriers (2 mg/mL). FLQY2 significant precipitate was observed due to the supersaturation of the drug in the aqueous solution. The mixture including PEG4000, PEG6000, PVP Va64, HS 15, and P407 showed crystallization and precipitation (Fig. 1B). But the saturated concentration of FLQY2 in Soluplus® maintained from 51.60 µg/mL at 5 min to 48.76 µg/mL at 8 h (Fig. 1B). In conclusion, Soluplus^®^ was considered the best carrier since it improved the solubility of FLQY2 and inhibited crystallization.

The dissolution of SD is a complex process involving multiple mechanisms, and the involvement of polymer-carriers can affect the dissolution behavior. The screening of drug-carrier ratios was shown in Fig. 1C. The amount of the dissolved FLQY2 increased with the addition of Soluplus^®^ from 1:5 to 1:20 but decreased when above 1:20. When the proportion was 1:5, the quantity of drugs in the molecularly dispersed form was relatively limited due to the reduced intermolecular distance and the small number of polymer-carriers. There was a risk of phase separation or crystallization, accounting for the reduced saturation solubility [35]. The saturation solubility of FLQY2 increased when the ratio reached 1:20. The saturation solubilities of the FLQY2-DM and FLQY2-PM groups were significantly lower than that of the FLQY2-SD group at the same drug-carrier ratio, indicating the effectiveness of the SDs. FLQY2-PM power prepared with the physical grinding mixture made a small amount of dissolved FLQY2. The optimal drug-carrier weight ratio was between 1:15 and 1:20, and 1:15 was chosen for the following studies.

### Characterization and in vitro release

**Fig. 2:**
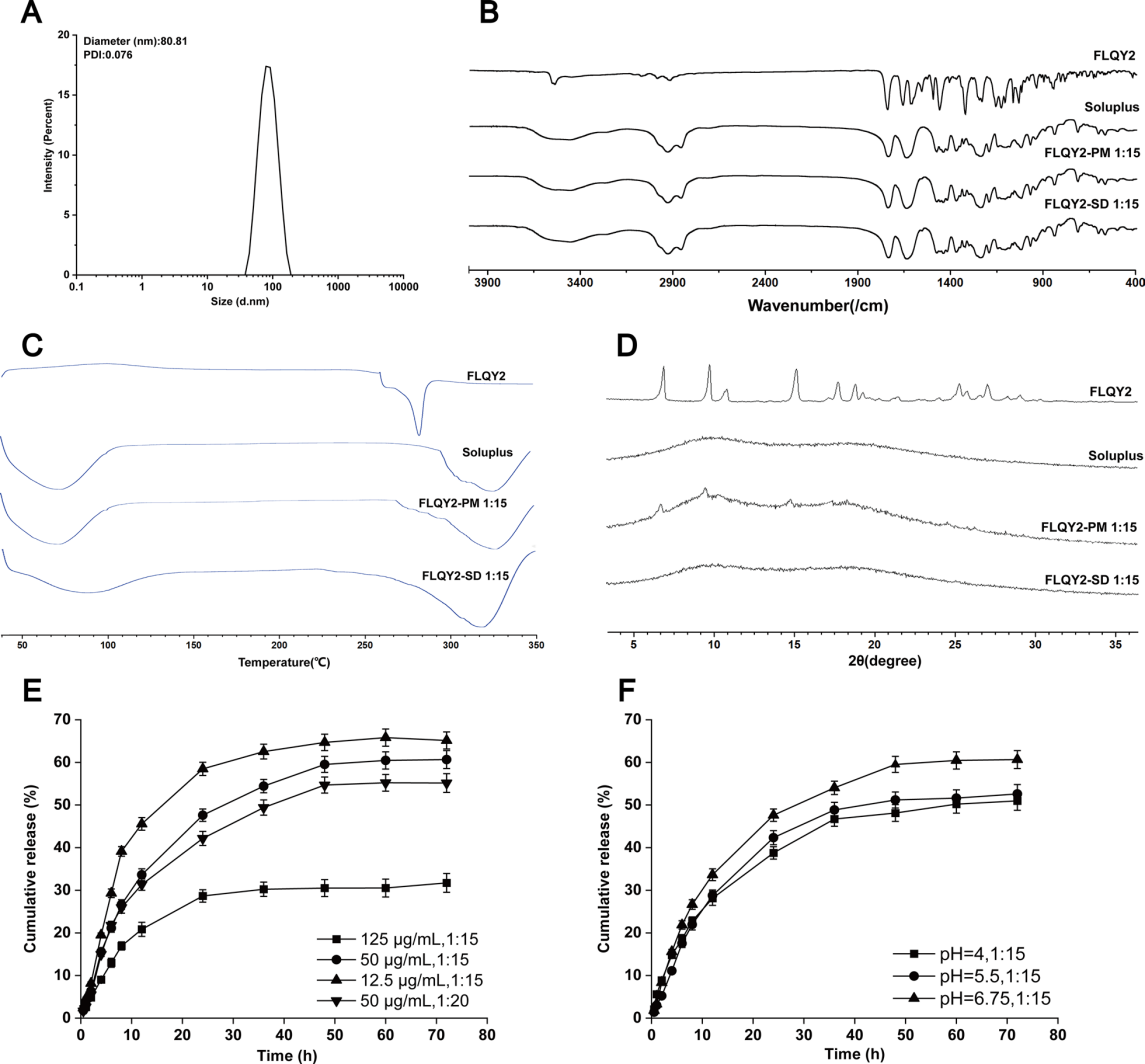
**A** Particle size and PDI of FLQY2-SD; **B** IR, **C** DSC, **D** XRD spectra of FLQY2-SD, FLQY2-PM, FLQY2, and Soluplus^**®**^; Drug release from FLQY2-SD in vitro at **E** various initial concentrations, weight ratios, and **F** pH of media

The prepared FLQY2-SD was characterized, and the results were shown in Fig. 2. The average particle size of FLQY2-SD micelles was 80.81 ± 2.1 nm with a PDI of 0.076, which implied a narrow particle size distribution and uniformity (Fig. 2A). The contents of FLQY2 in FLQY2-PM (1:15) were very low, moreover, the IR spectrum of Soluplus^**®**^ showed similar characteristics to the spectrum of FLQY2 in Fig. 2B, so the obvious difference could not be seen from the IR of the Soluplus, FLQY2-PM, and FLQY2-SD. The same phenomenon happened in DSC analysis in Fig. 2C. Pure FLQY2 exhibited sharp melting endotherms at 280 °C, corresponding to its crystalline nature. Although the crystalline FLQY2 was still present in FLQY2-PM power prepared with the physical grinding mixture, however the obvious endothermic peak at 280 °C did not appear and only the melting point of Soluplus was seen in the low contents of FLQY2 in FLQY2-PM. But the melting point in FLQY2-SD has shifted compared with only Soluplus, which indicated the interaction between FLQY2 and Soluplus. Although the contents of FLQY2 in FLQY2-PM (1:15) were very low, some sharp characteristic peaks of FLQY2 crystal in FLQY2-PM were found using XRD in Fig. 2D. FLQY2-SD, like Soluplus^®^, showed no characteristic peaks, which indicated that FLQY2 interacted with Soluplus^**®**^ and existed in an amorphous form. As well as if the interaction between FLQY2 and Soluplus^®^ was destroyed by organic solvents or unlimited dilution, FLQY2 would form immediately the crystal to precipitate, which could not be detected by HPLC.

The release behavior of SDs is closely related to pharmacodynamics. The effect of different initial concentrations, drug-carrier ratios, and pH on the drug in vitro release was investigated in Fig. 2E and F. The burst release of FLQY2-SD micelles in the first 8 h was approximately linear with time and slowed down after 8 h, reflecting the change in thermodynamic stability. The release rate of the “50 μg/mL, 1:15” group appeared to be faster than that of the more carrier group “50 μg/mL, 1:20”. The lowest release rate was observed for the “125 μg/mL, 1:15” group, and the low initial concentration increased the release rate. The different pH media were selected for release testing, and the acetate buffers at pH 4 and pH 5.5, and phosphate buffer at pH 6.75 had little effect on drug release from FLQY2-SD micelles in Fig. 2F. So the ratio of drug-carrier and the initial concentration were crucial factors for drug release.

### In vitro anticancer activity and cellular uptake assay

**Fig. 3:**
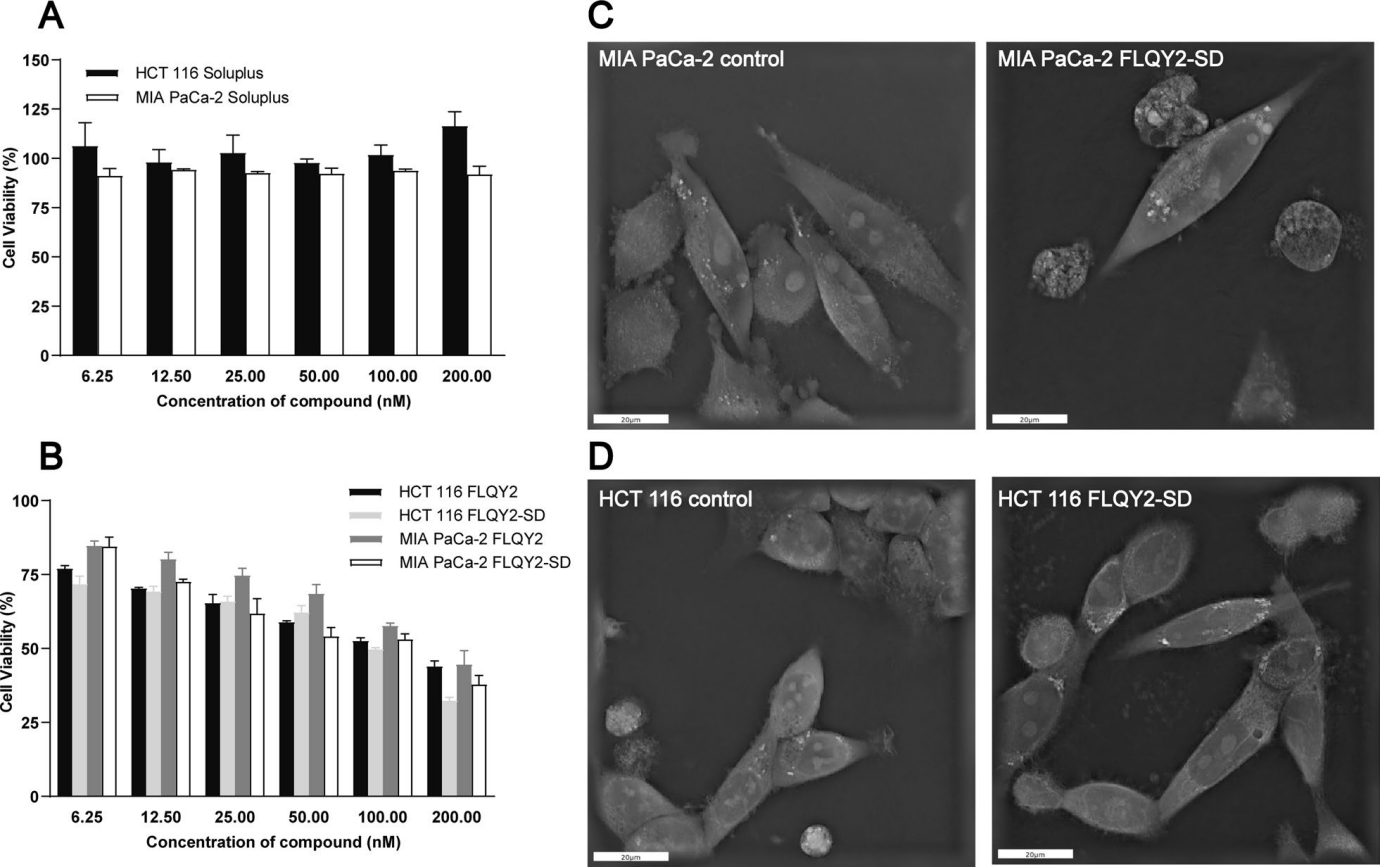
In vitro antitumor activity of **A** Soluplus^**®**^, **B** FLQY2, and FLQY2-SD in HCT 116 and MIA PaCa-2 cells (*p < 0.05; **p < 0.01; ***p < 0.001). The morphology images of **C** HCT 116 and **D** MIA PaCa-2 cell lines treated with FLQY2-SD or control were taken by a Nanolive 3D Cell Explorer (60 ×). The scale bars were 20 μm

HCT 116 and MIA PaCa-2 cell lines were selected for MTT assay. The cell viability exceeded 91.0 ± 3.8% after administration of the blank Soluplus^**®**^, indicating that Soluplus^**®**^ was a non-toxic drug delivery vehicle and biocompatible (Fig. 3A). Both FLQY2 and FLQY2-SD exhibited dose-dependent toxicity against the two cells, and no significant differences were found between these two treatments (Fig. 3B). Soluplus^®^ did not hinder the potent cytotoxic effect of FLQY2 in the range of the low administered concentrations. Figure 3C and D showed morphological changes in the cells before and after drug administration using a 3D tomography microscope (60** ×**). Untreated HCT 116 and MIA PaCa-2 cells exhibited an attached epithelial shape, while MIA PaCa-2 cells additionally had floating rounded cells. After treatment with FLQY2-SD for 72 h, some cells showed apoptotic-like morphological changes, such as shrinkage, nuclear fragmentation, and chromatin condensation, consistent with the literature [36, 37]. MTT assays performed with 1 μM of FLQY2/FLQY2-SD administered for 2 h showed cell viability exceeding 92.5 ± 7.6%, demonstrating that this time and concentration allowed for non-toxic and harmless cellular uptake assay.

**Fig. 4:**
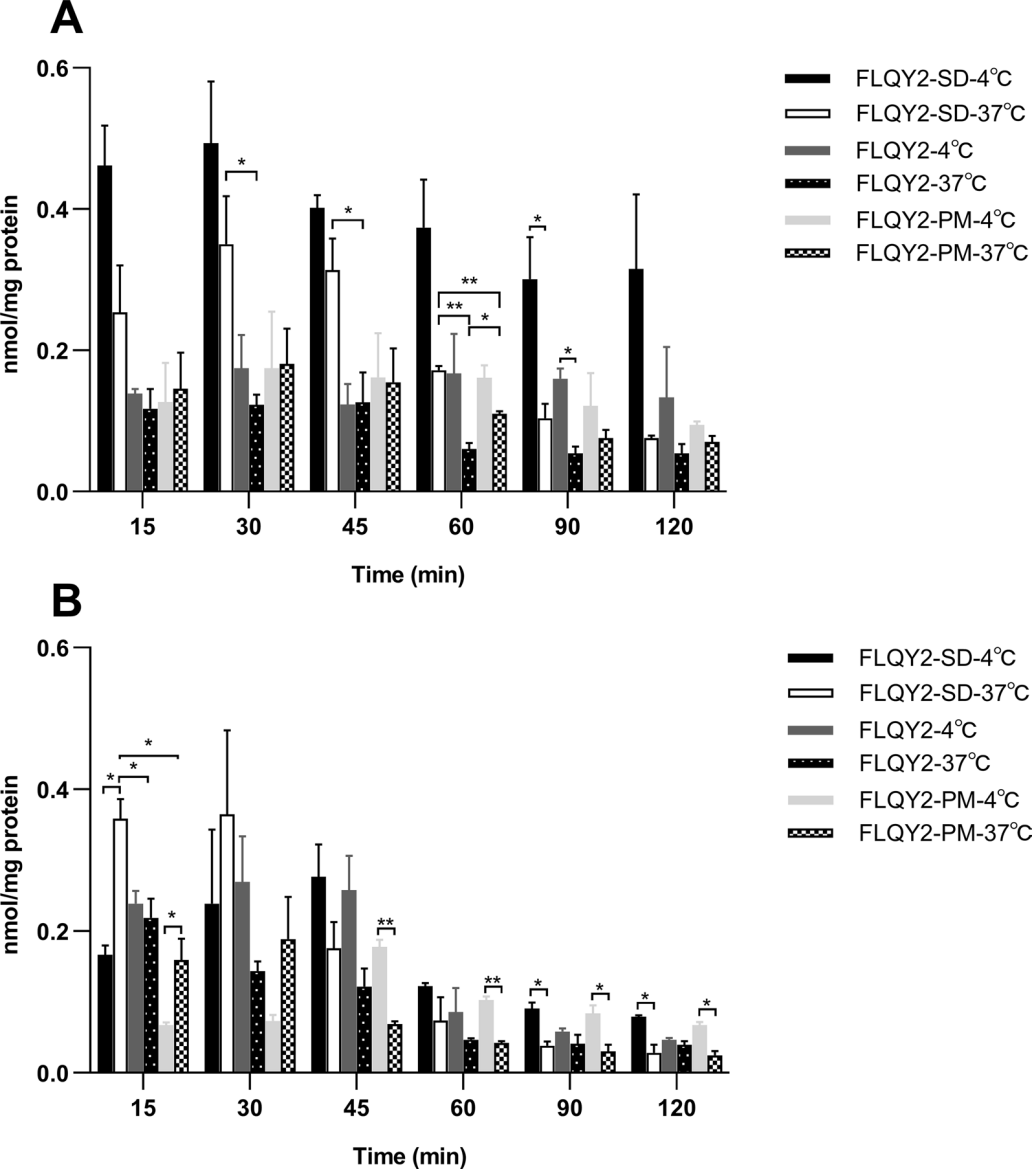
Cellular uptake of FLQY2/FLQY2-SD/FLQY2-PM in **A** HCT 116 and **B** MIA PaCa-2 cells

Cellular uptake was a dynamic process in which the effects of the carrier, temperature, and time on the uptake of FLQY2 were evaluated. As shown in Fig. 4A, internalization of FLQY2/FLQY2-SD/FLQY2-PM in both cells was time-dependent and driven mainly by passive diffusion, reaching maximum levels at 15–30 min at 37 °C. The content then decreased with time due to the efflux and the lactone ring hydrolysis of CPT analogs. Notably, the FLQY2-SD group showed faster uptake efficiency and a higher degree of internalization within 30 min, which related to the effectiveness of the SDs. Based on the assumption that the primary absorption mechanism is passive diffusion of free drugs, earlier studies on some copolymers noted that increasing partitioning into micelles would reduce the free fraction of drugs and their accumulation in the cells [38–40]. Soluplus^®^ serves as a polymer carrier, allowing polymeric micelles to form upon dissolution. However, Zhu’s group noted that no saturated solutions were formed in Soluplus^®^ or mixed micelles loaded with a low concentration of coumarin-6, and there were no significant cellular uptake differences among them [33]. The internalization of FLQY2 in HCT 116 cells at 4 °C was significantly higher than those at 37 °C. Temperature contributes to energy consumption and activity of transporter protein, and for passive diffusion, temperature also affects the partition/distribution coefficient and passive transcellular (paracellular) permeation [41]. Interestingly, the internalization of FLQY2-SD and FLQY2-PM at 4 °C was smaller than that of the 37 °C groups at 15 and 30 min in MIA PaCa-2 cells. Multiple transport mechanisms might coexist, such as the presence of energy-dependent endocytosis [42]. Results using chemical endocytosis inhibitors showed a significant reduction in cellular uptake of MβCD with simvastatin, which inhibited caveolae and clathrin-mediated pathways via cholesterol depletion (Additional file 1: Fig. S1). Reduced internalization with Nystatin suggested the involvement of caveolae-mediated pathways. However, the results of temperature and endocytosis inhibitors were correlated in MIA PaCa-2 cells, but not in HCT 116 cells. As Gaucher stated in her article, the exact mechanism controlling micelle permeation cannot be determined because adding endocytosis inhibitors and changing the temperature would produce contradictory results [43].

### Pharmacokinetics, tissue distribution, and excretion study

**Fig. 5:**
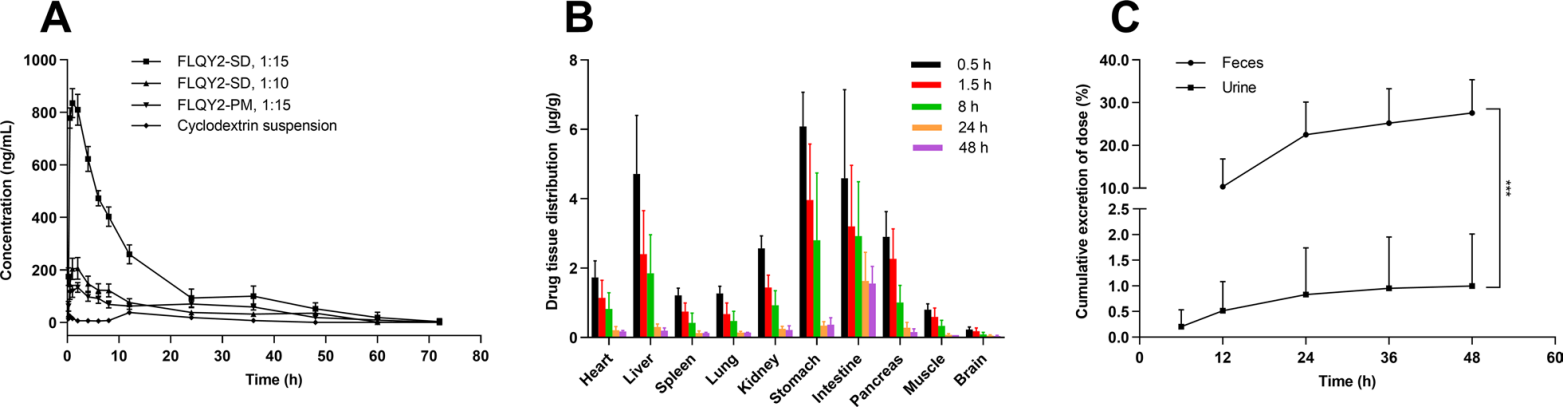
**A** Plasma FLQY2 concentration–time curve following oral administration of 8 mg/kg FLQY2 in different formulations. **B** Tissue distribution. **C** Accumulation and excretion in feces and urine after oral administration of 4 mg/kg FLQY2-SD

Female Sprague–Dawley rats were orally administered FLQY2 in different formulations, and the blood was taken through the eyeball of rats at the right time. The concentrations of FLQY2 in blood were measured by HPLC and analyzed by PK solver software using noncompartmental analysis. Figure 5A showed the pharmacokinetic curves, and Table 1 listed the pharmacokinetic parameters. T_max_, T_1/2,_ and MRT of all formulations were within 1–2 h, above 10 h, and above 15 h, respectively, implying FLQY2 in these four formulations was rapidly absorbed and slowly eliminated. AUC_0-72_ h of the “cyclodextrin suspension” group was the least effective (875.6 ± 58.6 h ng/mL), but the “FLQY2-SD, 1:15” group had the best absorption property (10,820.2 ± 936.3 h ng/mL) among these formulations with a 12.3-fold increase in bioavailability. Moreover, the bioavailability of the “FLQY2-SD, 1:15” group was 3.3-fold higher than that of the “FLQY2-PM, 1:15” group and 3.9-fold higher than that of the “FLQY2-SD, 1:10” group, elucidating “FLQY2-SD, 1:15” was the best for absorption enhancement. The solubility of FLQY2-SD depended on the degree of dissolution, which was influenced by the ratio between the drug and Soluplus^®^. When complexation with cyclodextrins increases the apparent solubility, the membrane/aqueous partitioning coefficient reduces, thus failing to bypass the solubility-permeability trade-off [23, 44]. On the contrary, supersaturation via the amorphous form does not affect the coefficient [45].

**Table 1:** Pharmacokinetic parameters of 8 mg/kg FLQY2 in different formulations after intragastric administration

Parameters (unit)	FLQY2-SD, 1:15	FLQY2-SD, 1:10	FLQY2-PM, 1:15	Cyclodextrin suspension
C_max_ (ng/mL)	835.0 ± 53.6^a^	206.3 ± 40.9^a^	133.2 ± 18.6^a^	37.9 ± 4.9
T_max_ (h)	1.3 ± 0.5	1.7 ± 0.5	1.7 ± 0.5	1(12)
MRT (h)	15.1 ± 1.4	19.69 ± 3.2	21.4 ± 2.7	25.4 ± 3.4
T_1/2_ (h)	10.0 ± 1.2	10.2 ± 1.6	12.4 ± 2.1	10.3 ± 1.4
AUC_0-72_ h (h·ng/mL)	10820.2 ± 936.3^a^	3507.8 ± 388.8^a^	3242.3 ± 353.2^a^	875.6 ± 58.6

^a^Represents

p < 0.0001, compared with the cyclodextrin suspension group

Tissue distribution and excretion of FLQY2-SD were further assessed in male and female Sprague–Dawley rats. As exemplified in Fig. 5B, FLQY2 was rapidly distributed to the tissues from the blood circulation. The maximum concentration of FLQY2 was 6.08 ± 0.98 μg/g in the stomach at 0.5 h, followed by the liver, intestine, and pancreas with concentrations of 4.71 ± 1.68 μg/g, 4.58 ± 2.55 μg/g, and 2.90 ± 0.72 μg/g. In addition, the accumulation of FLQY2 ranked in the following order (Additional file 1: Table S1): intestine  > stomach  > liver  > pancreas  > kidney  > heart  > lung  > spleen  > muscle  > brain. Compared to the previous distribution studies of FLQY2 cyclodextrin suspension [13], the improved FLQY2 accumulation in the stomach, intestine, and pancreas. The accumulation in the liver and kidney played a major role in clearance. FLQY2-SD was slowly eliminated, with a half-life of 29.09 ± 8.82 h in the intestine, demonstrating long-acting and slow-release characteristics.

Figure 5C showed the excretion of FLQY2 in rats over 48 h. 38.60% of the administered dose of FLQY2 was excreted through the primary fecal route (37.60%) and the secondary urinary route (1%). The excretion of FLQY2 occurred mainly in the first 24 h when 80.65 ± 5.84% of the excreted FLQY2 was already excreted from the feces. Overall, the elimination of FLQY2 may include prototype excretion, with fecal elimination being the primary pathway.

### Identification of metabolites and metabolic pathways of FLQY2

**Fig. 6:**
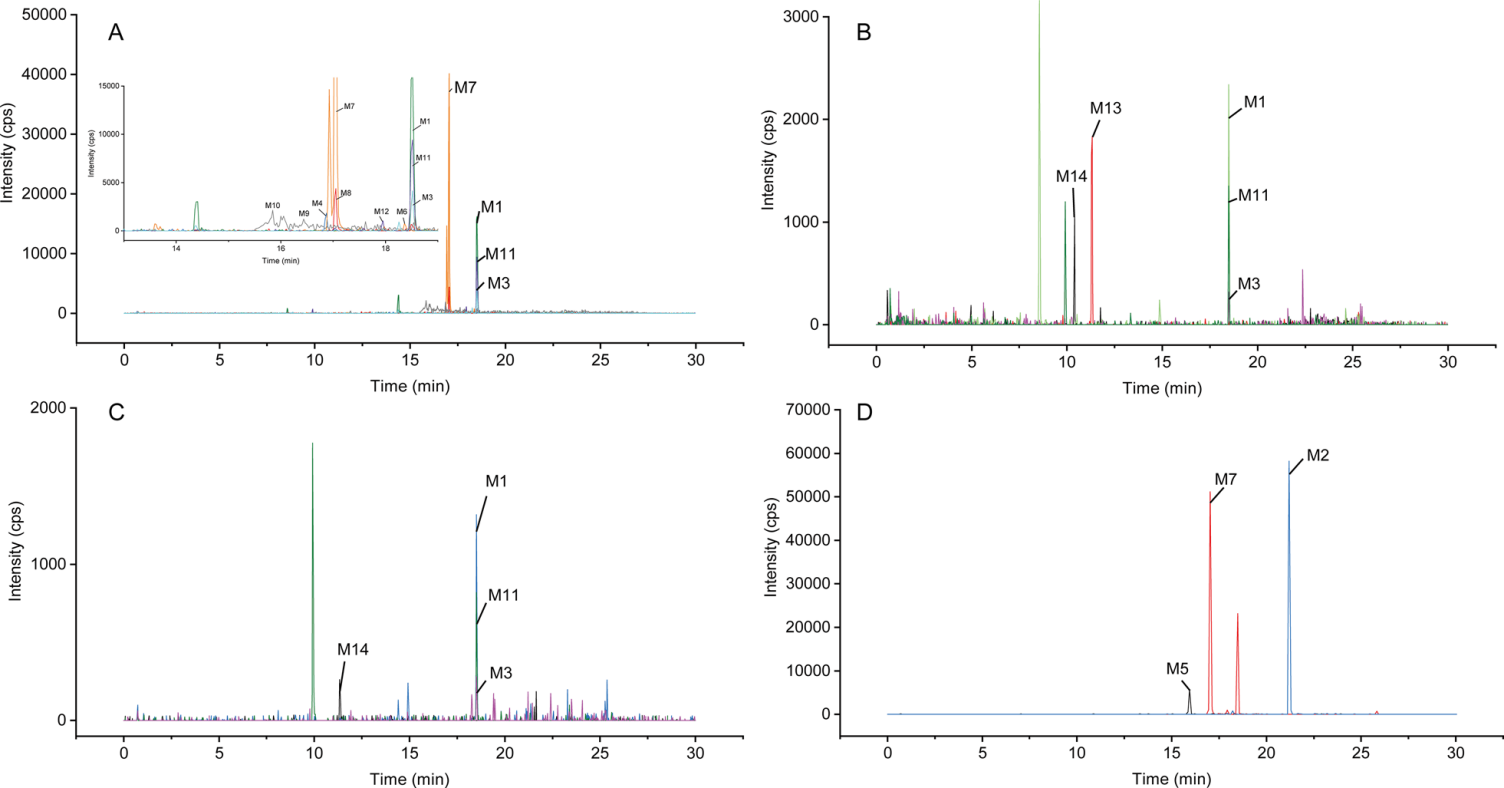
Combined extracted ion chromatograms of the metabolites from **A** ESI−, rat feces, **B** ESI−, rat urine, **C** ESI−, rat plasma, **D** ESI + , rat feces

**Fig. 7:**
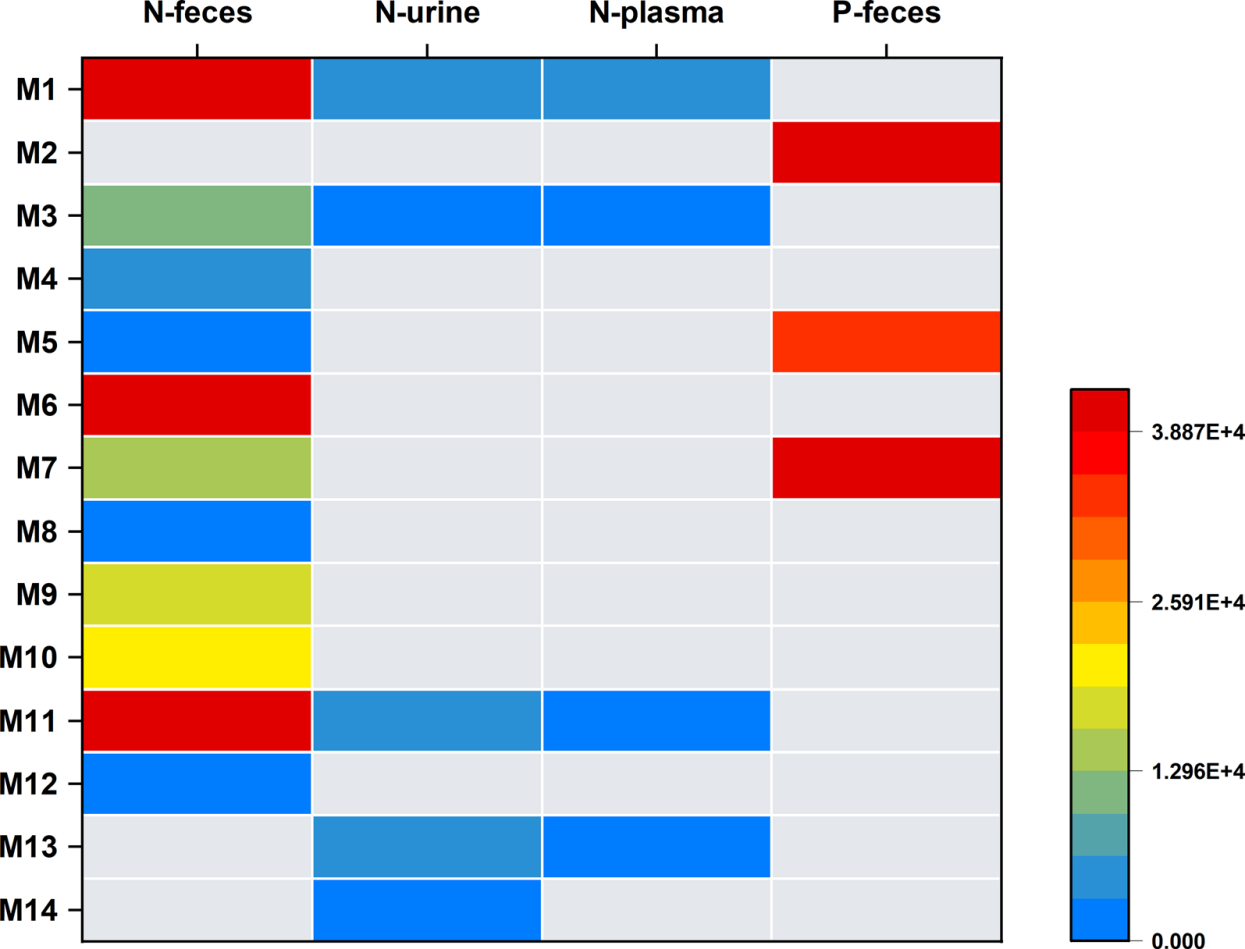
Heatmap of peak areas of the metabolites in different biological samples. While gray indicates that no metabolites were detected in the sample, blue and red represent low and higher peak areas, respectively

**Fig. 8:**
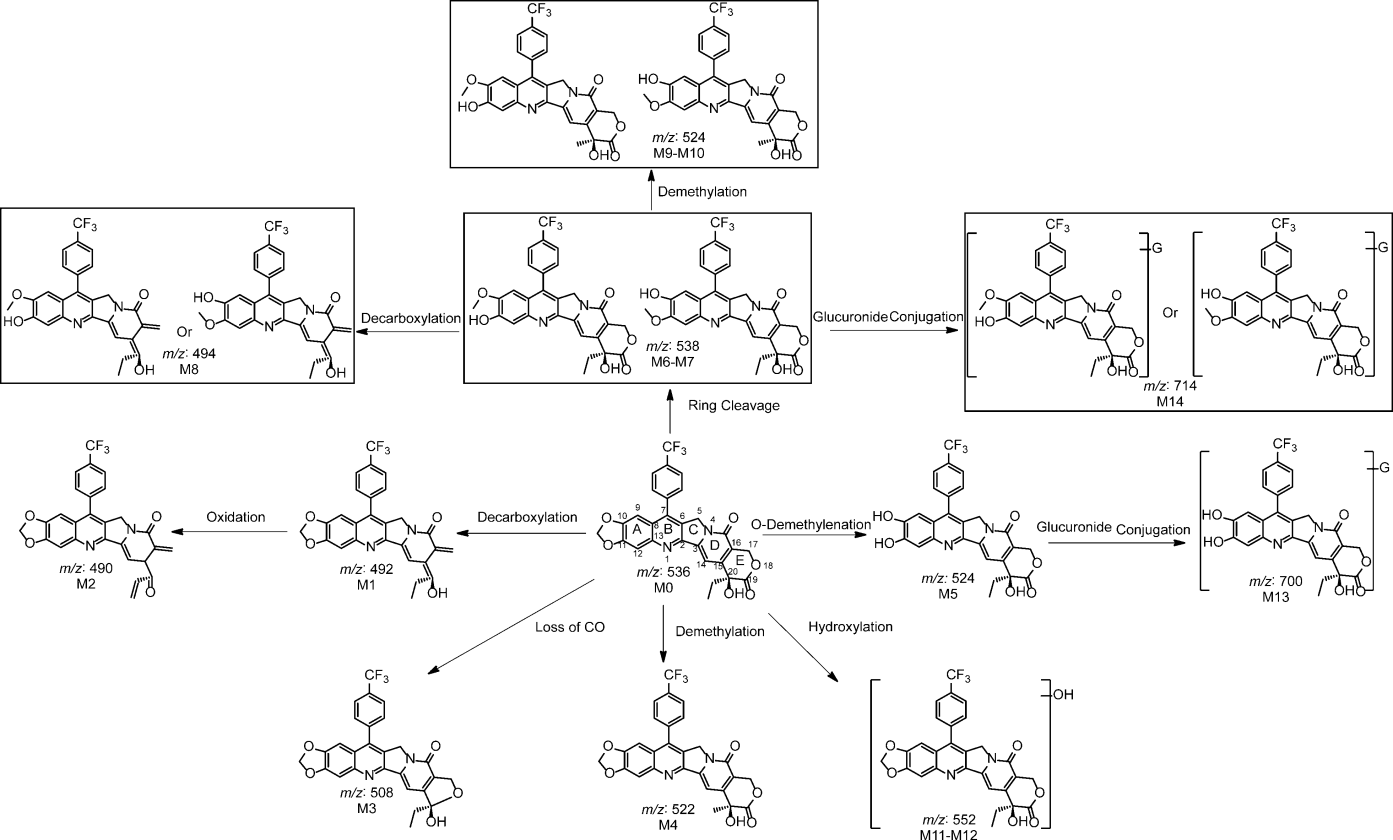
Proposed metabolic pathways of FLQY2 in rats

To further elucidate the disposition of the drug in vivo, the identification of metabolites was studied. All the bio-samples were analyzed using UPLC-TripleTOF^™^ 5600 in both positive and negative ion modes. The elemental compositions, selected ions, retention times, predicated masses, measured masses, errors, sources, and MS^2^ fragmental ions of the metabolites were summarized in Additional file 1: Table S2-S3. And the MS/MS details were listed in Additional File 1: Fig. S2-S4. To provide a metabolite profile, the merged extracted ion chromatograms of the identified metabolites in plasma, urine, or feces were displayed in Fig. 6. The 14 metabolites were discovered, including phase I metabolites (M1-M12) and phase II conjugation products (M13, M14), and all of them were identified for the first time. M7 (cleavage of the dioxolane ring) was the major metabolite detected in feces in the negative ion mode, while M2 (oxidized after decarboxylation) and M7 were the primary metabolites in the positive ion mode. The details were shown in a heatmap (Fig. 7). Based on the identified metabolites, the proposed pathways of FLQY2 were presented in Fig. 8. The first pathway was dioxolane ring cleavage, forming M6 and M7, which then underwent decarboxylation (M8), demethylation (M9–M10), and glucuronide conjugation (M14). The production of decarboxylation metabolite M1 was the second pathway, followed by oxidation to produce M2. The third route involved O-demethylenation at the dioxolane ring (M5), followed by glucuronide conjugation (M13). Loss of CO (M3), demethylation (M4), and oxidation (M11–M12) were other pathways.

### Anticancer activity in vivo

**Fig. 9:**
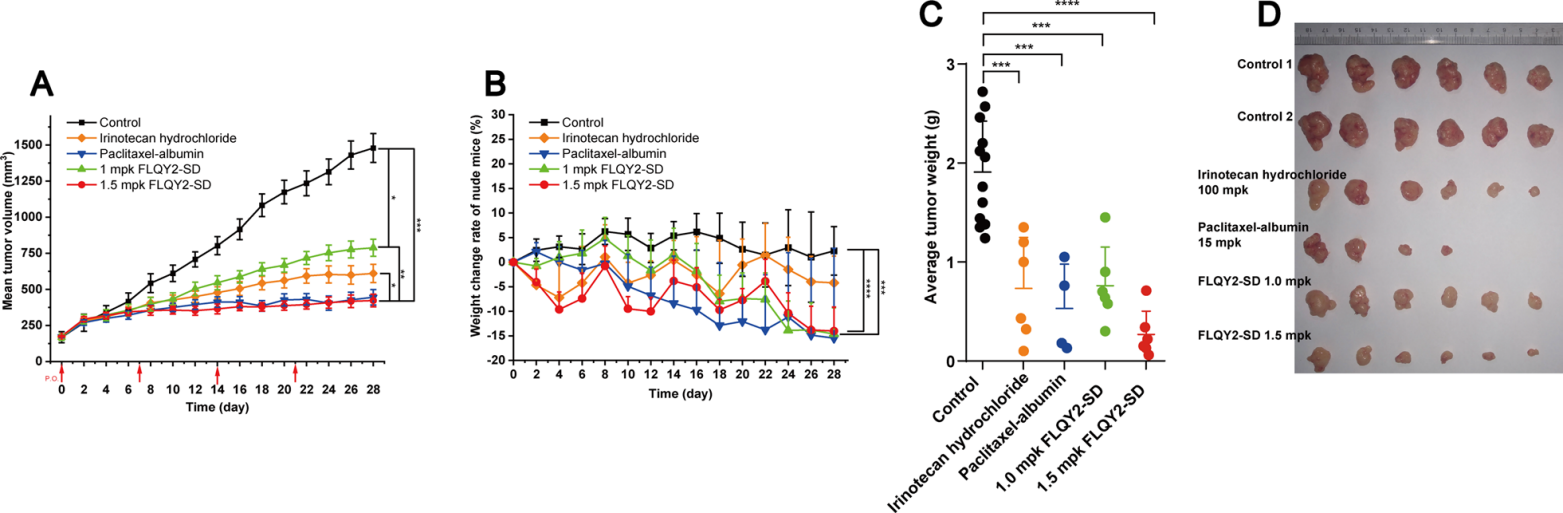
In vivo anticancer efficiency of FLQY2 (1.5 mpk, p.o./QW), FLQY2 (1 mpk, p.o./QW), Irinotecan hydrochloride (100 mpk, i.p./QW), Paclitaxel-albumin (15 mpk, i.v./Q4D) and saline (p.o.): **A** mean tumor volume, **B** weight change rate, **C** average tumor weight, and **D** photo of resected tumors

The antitumor activity in vivo of FLQY2-SD was evaluated on HT-29 tumor-bearing mice. 1.5 mpk and 1 mpk of FLQY2-SD were administered to mice with Irinotecan hydrochloride (200 mpk) and Paclitaxel-albumin (15 mpk) as positive references. Figure 9A showed that 1.5 mpk FLQY2-SD had comparable antitumor activity to Paclitaxel-albumin. Compared to the blank group (p < 0.001) and Irinotecan hydrochloride group (p < 0.01), 1.5 mpk FLQY2-SD effectively prevented tumor growth. The relative body weight of mice, a key indicator for assessing systemic toxicity, was decreased within 2–4 days after FLQY2-SD administration while showing significant recovery after 5–7 days. However, the Paclitaxel group did not show a recovery of the decreased body weight, and 2 mice were sacrificed (Fig. 9B). After 28 days, the remaining mice were sacrificed, and the tumors were removed and weighed, as shown in Fig. 9C and D. The average tumor weight was in the order of 1.5 mpk FLQY2-SD  < Paclitaxel-albumin  < Irinotecan  < 1 mpk FLQY2-SD  < saline group. Moreover, 1.5 mpk FLQY2-SD showed an excellent TGI rate of 81.1%, similar to Paclitaxel-albumin (79.1%). The TGI of 1 mpk FLQY2-SD and Irinotecan was 52.7% and 66.5%, respectively. The article also reported that 9-NC-SD improved the solubility and permeability of 9-NC and owned a significantly higher TGI rate in ICR bearing S180 tumors [19]. In brief, FLQY2-SD exhibited low effective doses (1–1.5 mpk), low frequency (once a week), tolerable toxicity, and excellent antitumor activity than 15 mpk Paclitaxel and 100 mpk Irinotecan, suggesting that FLQY2-SD was a promising candidate formulation for colon cancer.

## Conclusions

In this project, FLQY2-SD was prepared by a solvent evaporation method. The optimal FLQY2-SD had a mean particle size of 80.81 ± 2.1 nm, a PDI of 0.076, saturated solubility of 5.32 ± 0.04 mg/mL, and a drug loading of 6.2 ± 0.2%. Characterization studies demonstrated that FLQY2 existed in SDs in an amorphous form. The FLQY2-SD micelles did not affect cytotoxicity or cellular uptake of FLQY2 in vitro. The bioavailability of FLQY2-SD was increased by 12.3-fold compared to the cyclodextrin suspension of FLQY2. FLQY2-SD was rapidly distributed from the blood circulation to the intestinal, stomach, liver, and pancreas tissues with long half-life periods (T_1/2_ > 10 h) and subsequently eliminated mainly by fecal excretion. The primary metabolic pathway was the cleavage of the dioxolane ring. Pharmacodynamic studies revealed that FLQY2-SD possessed excellent antitumor activity with a TGI of 81.1% compared with Paclitaxel-albumin and Irinotecan. Therefore, FLQY2-SD was a promising formulation, and SD could be used as a potential delivery system for camptothecin analogs.

## Supplementary Information

**Additional file 1: Figure S1**. Effect of endocytosis inhibitors on FLQY2-SD uptake by HCT 116 and MIA PaCa-2 cells.** Table S1**. Pharmacokinetic parameters in rat tissues after oral administration of 4 mg/kg FLQY2-SD.** Table S2**. Parameters of the metabolites detected using ultrahigh performance liquid chromatography coupled with quadrupole time-of-flight mass spectrometry in ESI −. **Table S3**. Parameters of the metabolites detected using ultrahigh performance liquid chromatography coupled with quadrupole time-of-flight mass spectrometry in ESI +. **Figure S2**. (A) Extracted ion chromatograms of FLQY2 (M0) in ESI +, (B) MS/MS spectrum, and the proposed fragmentation pathway of FLQY2 **Figure S3**. MS/MS spectrum and the proposed fragmentation pathways of (A) M1, (B) M2, (C) M3, (D) M4, (E) M5, (F) (G) M6 or M7 **Figure S4**. MS/MS spectrum and the proposed fragmentation pathways of (A) M8, (B) (C) M9 or M10, (D) M11 and M12, (E) M13, and (F) M14.

## Data Availability

All data generated or analyzed during this study are included in this published article and its supplementary information files.

## Abbreviations

CMC: Critical micelle concentration;
CPT: Camptothecin
DMEM: Dulbecco’s modified eagle medium
DMSO: Dimethylsulfoxide
DSC: Differential scanning calorimetry
ESI: Electrospray ionization
FBS: Fetal bovine serum
HPLC: High performance liquid chromatography
IC_50_: Half maximal inhibitory concentration
IDA: Information dependent acquisition
i.p.: Intraperitoneal injection
IR: Infrared spectral analysis
IS: Internal standard
i.v.: Intravenous injection
mpk: Milligrams per kilograms
MTT: 3-(4, 5-Dimethylthiazolyl-2)-2, 5-diphenyltetrazolium bromide
PDI: Polydispersity index
PM: Physical mixture
p.o.: Peros
Q4D: Quaque 4 days
QW: Quaque week
RTV: Relative tumor volume
SD: Solid dispersion
Soluplus^®^: Polyvinyl caprolactam-polyvinyl acetate-polyethylene glycol graft copolymer
TGI: Tumor growth inhibition
TV: Tumor volume
UPLC-Q-TOF–MS: Ultra performance liquid chromatography-quadrupole-time of flight-mass spectrometry
XRD: X-ray diffraction
