# Supplementary material for: Preparation of a camptothecin analog FLQY2 self-micelle solid dispersion with improved solubility and bioavailability
Source: J Nanobiotechnology. 2022 Sep 5;20:402. doi: 10.1186/s12951-022-01596-2 (PMC9446799; doi:10.1186/s12951-022-01596-2)
Supplement: Supplementary file 1 — Additional file 1: Figure S1. Effect of endocytosis inhibitors on FLQY2-SD uptake by HCT 116 and MIA PaCa-2 cells. Table S1. Pharmacokinetic parameters in rat tissues after oral administration of 4 mg/kg FLQY2-SD. Table S2. Parameters of the metabolites detected using ultrahigh performance liquid chromatography coupled with quadrupole time-of-flight mass spectrometry in ESI −. Table S3. Parameters of the metabolites detected using ultrahigh performance liquid chromatography coupled with quadrupole time-of-flight mass spectrometry in ESI +. Figure S2. (A) Extracted ion chromatograms of FLQY2 (M0) in ESI +, (B) MS/MS spectrum, and the proposed fragmentation pathway of FLQY2 Figure S3. MS/MS spectrum and the proposed fragmentation pathways of (A) M1, (B) M2, (C) M3, (D) M4, (E) M5, (F) (G) M6 or M7 Figure S4. MS/MS spectrum and the proposed fragmentation pathways of (A) M8, (B) (C) M9 or M10, (D) M11 and M12, (E) M13, and (F) M14. [file 12951_2022_1596_MOESM1_ESM.docx]

**Preparation of a camptothecin analog FLQY2 self-micelle solid dispersion with improved solubility and bioavailability**

**Yi Wang, Wenchao Wang^#^, Endian Yu, Wenya Zhuang, Xuanrong Sun, Hong Wang, Qingyong Li**^*^

^*^Correspondence: li_qingyong@126.com

College of Pharmaceutical Sciences, Zhejiang University of Technology, Hangzhou, China

Full lists of author information are available at the end of the article

^#^Co-first author

**Methods**

**1. Materials**

FLQY2-SD was prepared by our laboratory. Nystatin, Chlorpromazine, and Methyl-β-cyclodextrin (MβCD) were purchased from Help Easy Co., Ltd (Hangzhou, China). Simvastatin was obtained from MERYER Co., Ltd (Shanghai, China).

**2. Cellular uptake assay**

Nystatin (25 µmol/L), Chlorpromazine (10 mg/L), or MβCD (10 g/L) + Simvastatin (1 mg/L) was were preincubated for 30min, respectively. FLQY2-SD micelles solution (1 µmol/L) was added to the plates and then incubated for 30 min at 37℃. The cells were rinsed, collected, and lysed with the medium removed after three freeze-thaw cycles. The concentration of protein was determined by the Coomassie Brilliant Blue method, while the concentration of FLQY2 was determined by HPLC.

**
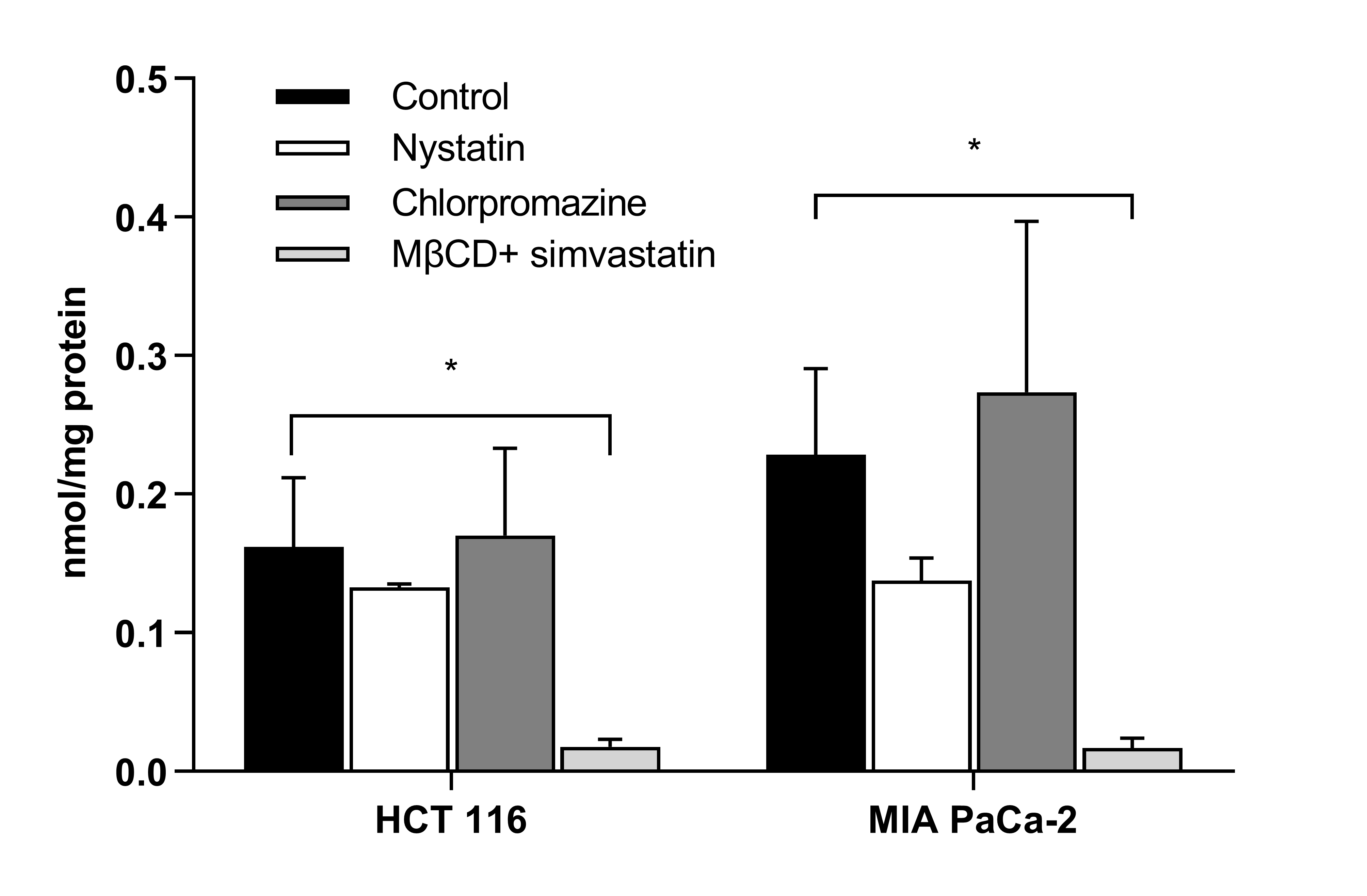
**

**Fig. S1**. Effect of endocytosis inhibitors on FLQY2-SD uptake by HCT 116 and MIA PaCa-2 cells.

| Parameters | C_max_ (ng/mL) | T_max_ (h) | T_1/2_ (h) | AUC_0-48_ (h·ng/mL) |
| --- | --- | --- | --- | --- |
| Heart | 132.89 ± 42.29 | 0.5 | 15.68 ± 3.74 | 1634.88 ± 655.25 |
| Liver | 431.34 ± 152.53 | 0.5 | 11.58 ± 1.53 | 3759.47 ± 1854.96 |
| Spleen | 105.34 ± 16.87 | 0.5 | 16.12 ± 1.02 | 1041.61 ± 498.75 |
| lung | 111.48 ± 17.92 | 0.5 | 19.45 ± 6.53 | 1153.57 ± 439.50 |
| Kidney | 231.66 ± 31.06 | 0.5 | 14.66 ± 2.04 | 2256.50 ± 753.91 |
| Stomach | 497.33 ± 87.98 | 0.5 | 11.67 ± 0.55 | 5107.10 ± 2742.33 |
| Intestine | 411.95 ± 221.39 | 0.5 | 29.09 ± 8.82 | 8489.25 ± 4744.09 |
| Pancreas | 259.74 ± 58.56 | 0.5 | 11.45 ± 0.92 | 2655.18 ± 1163.34 |
| Muscle | 58.39 ± 15.12 | 0.5 | 8.68 ± 2.03 | 607.88 ± 316.92 |
| Brain | 15.23 ± 4.85 | 0.5 | 27.31 ± 10.38 | 241.34 ± 116.76 |

**Table S1**. Pharmacokinetic parameters in rat tissues after oral administration of 4 mg/kg FLQY2-SD

|  | Identification |  | Decarboxylation | Loss of CO | Ring Cleavage | Ring Cleavage | Ring Cleavage + Decarboxylation | Ring Cleavage + Demethylation | Ring Cleavage + Demethylation |
| --- | --- | --- | --- | --- | --- | --- | --- | --- | --- |
| MS/MS | ESI- | 491.1225，476.0984，463.1308， 435.0917，407.0992 | 476.0968，462.1213 | 489.8201，478.7950 | 493.1385，478.1142，450.1186 | 493.1379，478.1136，450.1192 | 478.1135，450.1191 | 505.1004，479.1226 | 479.1214 |
| [M-H]- | Source | F, U, P | F, U, P | F, U, P | F | F | F | F | F |
|  | Mass error | -0.56 | -1.08 | -3.98 | 2.14 | 2.14 | -3.20 | -1.15 | 0.38 |
|  | Measured mass | 535.112 | 491.1224 | 507.1188 | 537.1262 | 537.1262 | 493.1391 | 523.1123 | 523.1115 |
|  | Predicated mass | 535.1117 | 491.1219 | 507.1169 | 537.1274 | 537.1274 | 493.1375 | 523.1117 | 523.1117 |
|  | Retention time （min） | 18.481 | 18.502 | 18.524 | 18.508 | 17.044 | 17.044 | 16.463 | 15.836 |
|  | Elemental composition | C28H19F3N2O6 | C27H19F3N2O4 | C27H19F3N2O5 | C28H21F3N2O6 | C28H21F3N2O6 | C27H21F3N2O4 | C27H19F3N2O6 | C27H19F3N2O6 |
|  |  | M0 | M1 | M3 | M6 | M7 | M8 | M9 | M10 |

|  | Identification | Hydroxylation | Hydroxylation | O-Demethylenation+Glucuronide Conjugation | Ring Cleavage + Glucuronide Conjugation |
| --- | --- | --- | --- | --- | --- |
| MS/MS | ESI- | 507.1176，489.1071，479.1230 | 507.1177，489.1065，479.1227 | 654.7081，522.7525，478.7928 | 536.7586，492.7979 |
| [M-H]- | Source | F, U, P | F | U, P | U |
|  | Mass error | -2.70 | -4.88 | -5.61 | -0.24 |
|  | Measured mass | 551.1081 | 551.1093 | 699.1477 | 713.1596 |
|  | Predicated mass | 551.1066 | 551.1066 | 699.1439 | 713.1594 |
|  | Retention time （min） | 18.505 | 17.951 | 11.310 | 10.419 |
|  | Elemental composition | C28H19F3N2O7 | C28H19F3N2O7 | C33H27F3N2O12 | C34H29F3N2O12 |
|  |  | M11 | M12 | M13 | M14 |

**Table S2**. Parameters of the metabolites detected using ultrahigh performance liquid chromatography coupled with quadrupole time-of-flight mass spectrometry in ESI −

|  | Identification |  | Decarboxylation +Oxidation | O-Demethylenation | Ring Cleavage |
| --- | --- | --- | --- | --- | --- |
| MS/MS | ESI+ | 508.0885，465.1427，437.1125，409.1155 | 476.0977 | 481.1409，425.2285 | 495.1545，480.1307，452.1367，439.1270， |
| [M+H]+ | Source | F, U, P | F | F | F |
|  | Mass error | 0.28 | -0.67 | 1.43 | 0.93 |
|  | Measured mass | 537.1272 | 491.1222 | 525.1266 | 539.1425 |
|  | Predicated mass | 537.1273 | 491.1219 | 525.1274 | 539.1430 |
|  | Retention time （min） | 18.486 | 21.205 | 15.948 | 17.096 |
|  | Elemental composition | C28H19F3N2O6 | C27H17F3N2O4 | C27H19F3N2O6 | C28H21F3N2O6 |
|  |  | M0 | M2 | M5 | M7 |

**Table S3**. Parameters of the metabolites detected using ultrahigh performance liquid chromatography coupled with quadrupole time-of-flight mass spectrometry in ESI +


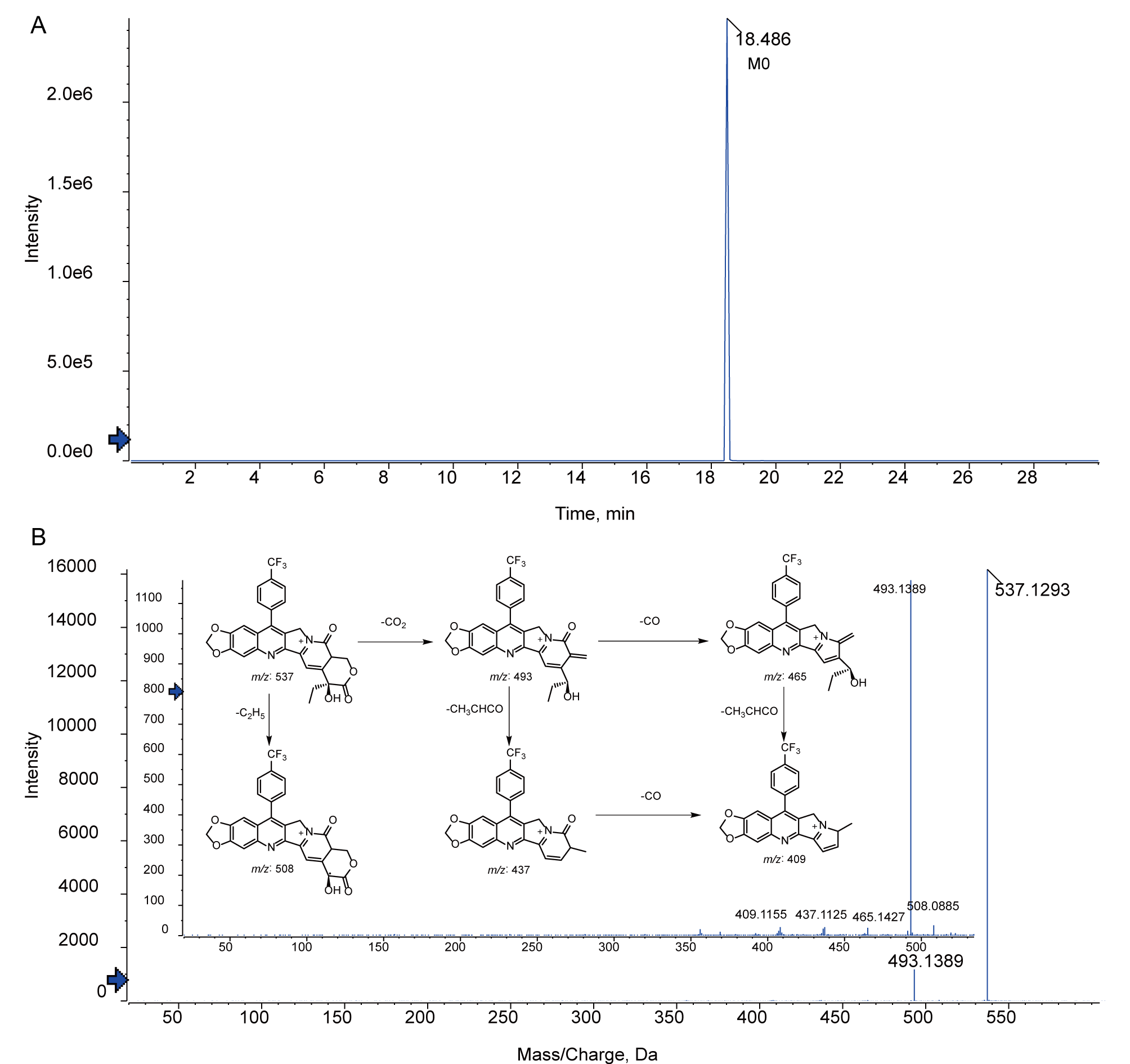


**Fig. S2**. (A) Extracted ion chromatograms of FLQY2 (M0) in ESI +, (B) MS/MS spectrum, and the proposed fragmentation pathway of FLQY2


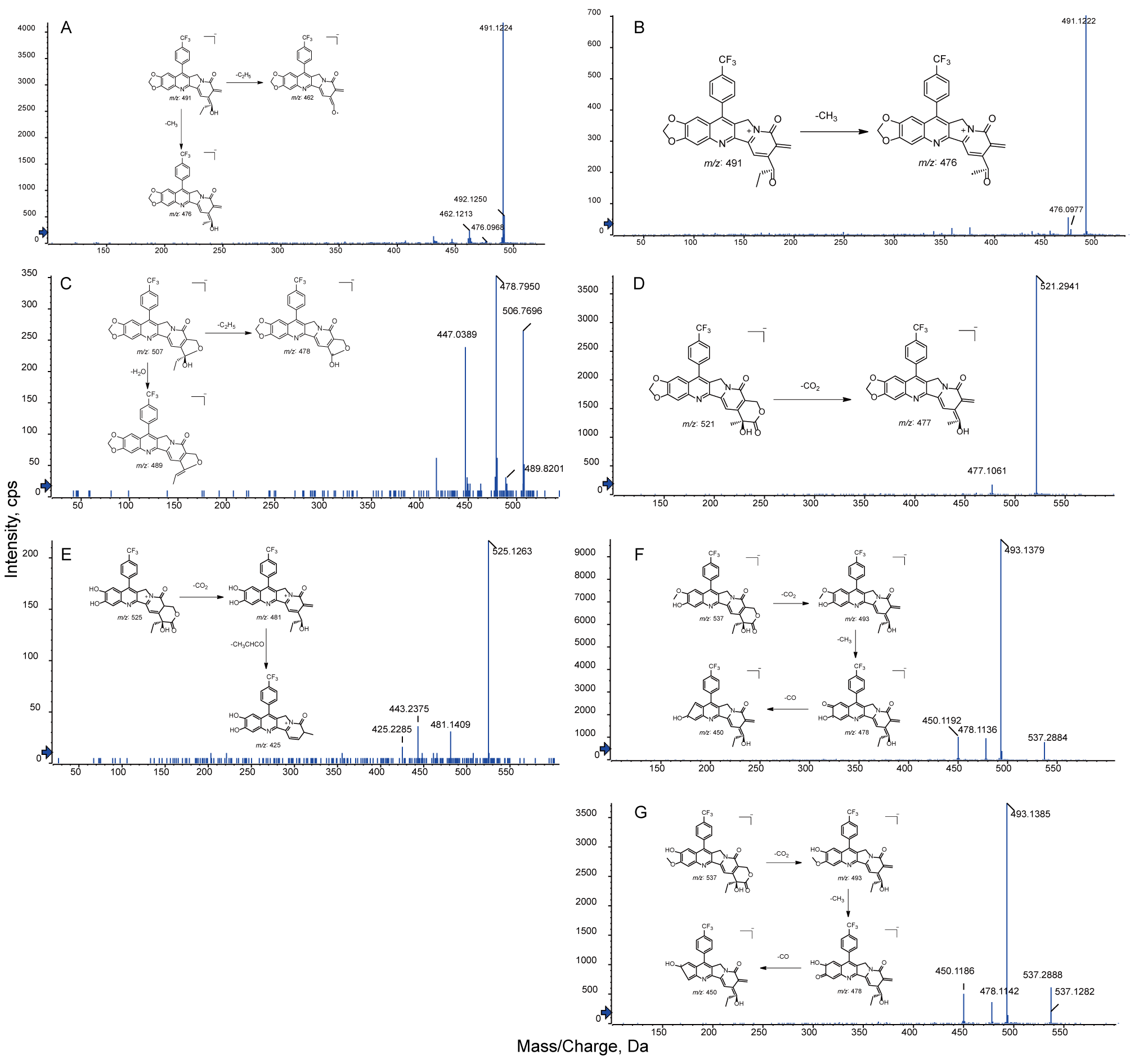


**Fig. S3**. MS/MS spectrum and the proposed fragmentation pathways of (A) M1, (B) M2, (C) M3, (D) M4, (E) M5, (F) (G) M6 or M7


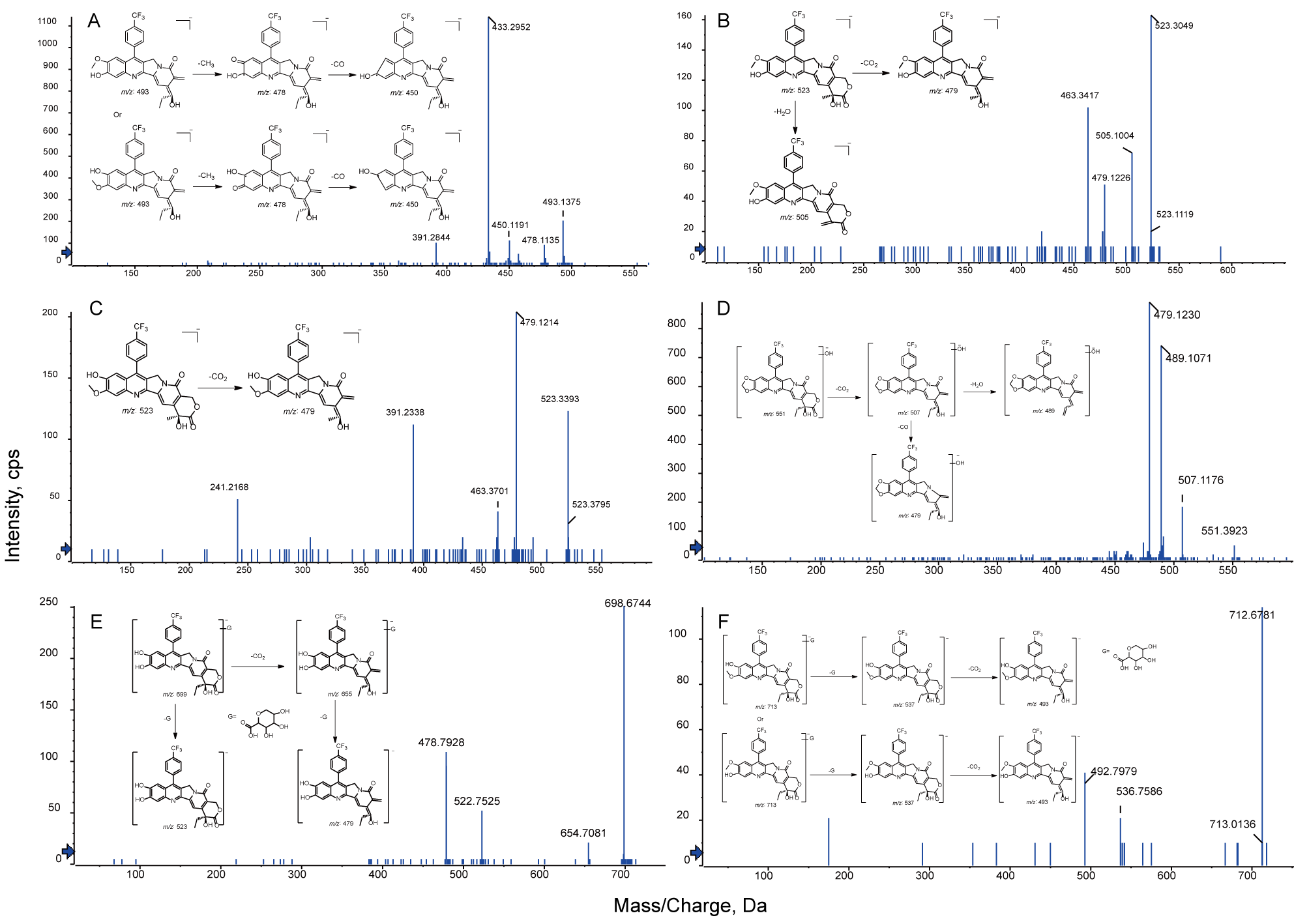


**Fig. S4**. MS/MS spectrum and the proposed fragmentation pathways of (A) M8, (B) (C) M9 or M10, (D) M11 and M12, (E) M13, (F) M14

**Identification of Metabolites of FLQY2**

**1. Characteristics of FLQY2 (M0) fragmentation**

Eluted at 18.486 min, the protonated ions of M0 (C_28_H_19_F_3_N_2_O_6_) were at *m/z* 537.1272 in the positive mode and 535.1120 in the negative mode. According to the MS/MS spectrum in ESI +, the product ions at *m/z* 493.1389 or *m/z* 508.0885 were attributed to the removal of CO_2_ at the lactone ring or the loss of C_2_H_5_. Additionally, the fragment ion of 437.1125 was derived from the loss of CH_3_CHCO by hydrogen rearrangement of the ion of 493.1389. The removal of CO at the D ring yielded the fragment ion at *m/z* 465.1427. The fragment ion at *m/z* 409.1155 was due to the loss of CO from the ion 437.1125 or the rearrangement of hydrogen from the ion 465.1427. The possible fragmentation patterns of FLQY2 were shown in Fig. S2.

**2. Characteristics of M1**

M1 (C_27_H_19_F_3_N_2_O_4_) with a molecular ion [M−H]^−^ at *m/z* 491.1224 was detected at 18.502 min, 44 Da lower than that of M0. As exhibited in Fig. S3(A), the MS/MS spectrum showed an ion at m/z 476.0968 owing to the breakage of CH_3_. Another fragment ion at *m/z* 462.1213 was probably generated by the loss of C_2_H_5_. M1 was considered a decarboxylated metabolite of the parent M0.

**3. Characteristics of M2**

Found at 21.255 min, the molecular ion of M2 (C_27_H_17_F_3_N_2_O_4_) was presumed to be *m/z* 491.1222 [M+H]^+^, which was 46 Da lower than that of M0 and 2 Da lower than that of M1. The indicative product ion displayed in Fig. S3(B) was at *m/z* 476.0977, which was caused by removing CH_3_. M1 might oxidize to produce metabolite M2.

**4. Characteristics of M3**

M3 (C_27_H_19_F_3_N_2_O_5_) had the molecular ion [M−H]^−^ at *m/z* 507.1186, 28 Da lower than that of M0, which was found at 18.524 min, The MS/MS spectrum in Fig. S3(C) revealed characteristic fragment ions at *m/z* 478.7950 and 489.7697, which were caused by the removal of C_2_H_5_ and H_2_O from ion 507. As a result, M3 was proposed as the decarbonylated metabolite of M0.

**5. Characteristics of M4**

Identified at 16.872 min, the molecular ion [M−H]^−^ of M4 (C_27_H_17_F_3_N_2_O_6_) was at *m/z* 521.0975, which was 14 Da lower than that of M0. As shown in Fig. S3(D), an indicative protonated ion appeared at *m/z* 477.1061 in the MS/MS spectrum, also 14 Da less than the product ion of M0 at *m/z* 491, indicating decarboxylation at the lactone ring took place. Thus, M4 was characterized as the demethylated metabolite of M0.

**6. Characteristics of M5**

With the molecular ion [M+H]^+^ at *m/z* 525.1266, M5 (C_27_H_19_F_3_N_2_O_6_) was detected at 15.948 min. According to the MS/MS spectrum in Fig. S3(E), the presence of fragment ion at *m/z* 481.1409, which was 12 Da less than that of M0, proved the absence of CO_2_. And then, the ion 425.2285 was produced by the rearrangement of hydrogen, which was 12 Da lower than the product ion of M0 at *m/z* 437. Therefore, M5 was attributed as the O-demethylenated product of M0.

**7. Characteristics of M6-M7**

M6 and M7 (C_28_H_21_F_3_N_2_O_6_) had the same molecular ion [M−H]^−^ at *m/z* 537.1262 in the negative mode, while only M7 had the molecular ion [M+H]^+^ at *m/z* 539.1425. And the retention times were 18.508 min and 17.044 min, respectively. Take the ESI − MS/MS spectrum of M6 in Fig. S3(F) or (G) for example, the product ion at *m/z* 493.1379 indicated that decarboxylation happened at the lactone moiety. The other product ion at *m/z* 478.1136 demonstrated the removal of CH_3_ at the C-10 or C-11 position, followed by decarbonylated probably occurring at the same position to give ion 450.1192. Therefore, we proposed M6 and M7 as ring cleavage metabolites of M0. However, the exact structural formula could not be distinguished from the present results.

**8. Characteristics of M8**

The molecular ion of M8 (C_27_H_2_1F_3_N_2_O_4_) was at *m/z* 493.1391 in the negative mode, which was 44 Da lower than that of M6/M7. And the retention time was 17.044 min. It was indicative that the fragment ion at *m/z* 478.1135 was formed by the breakage of CH_3_ shown in Fig. S4(A). The fragment ion at *m/z* 450.1191 demonstrated the loss of CO, which was similar to the fragmentation pattern of M6/M7 in the negative mode. Therefore, M8 was a decarboxylated metabolite of M6/M7.

**9. Characteristics of M9-M10**

The retention time of M9 and M10 (C_27_H_19_F_3_N_2_O_6_) were 16.463 and 15.836 min, and the molecular ions in the negative mode were *m/z* 523.1123 and 523.1115, which were 14 Da less than that of M6/M7, suggesting the occurrence of demethylation. The fragment ion with *m/z* 479.1226 was shown in Fig. S4(B) or (C), which was 14 Da less than that of decarboxylation ion M8, suggesting the loss of CO_2_ occurred at M9 or M10. The fragment ion *m/z* 505.1004 resulted from the loss of H_2_O. Therefore, M9/M10 was identified as the demethylated metabolite of M6/M7.

**10. Characteristics of M11-M12**

M11 and M12 (C_28_H_19_F_3_N_2_O_7_) had retention times of 18.505 min and 17.951 min. The fragment ion [M−H]^−^ *m/z* 551 was 16 Da more than that of M0, indicating the addition of O. As shown in Fig. S4(D), the MS/MS spectrum of M11 provided a characteristic fragment ion at *m/z* 507.1176, which was 16 Da larger than the decarboxylation product ion of M0 at *m/z* 491, indicating the decarboxylation also occurred in M11. Moreover, the product ions at *m/z* 479.1230 and 489.1071 were obtained from the ion *m/z* 507 by removing CO and H_2_O. The fragmentation patterns of M12 were the same as that of M11. As a result, we proposed that M0 hydroxylated yield M11 and M12, while the exact structural formula could not be determined yet.

**11. Characteristics of M13**

The molecular ion [M−H]^−^ of M13 (C_33_H_27_F_3_N_2_O_12_) was identified at *m/z* 699.1477, 176 Da more than that of M5. And the retention time of M13 in the chromatography was 11.310 min. A product ion with *m/z* 654.7801 was found in Fig. S4(E), probably due to the occurrence of decarboxylation. The fragment ion at *m/z* 522.7525 and 478.7928 demonstrated the loss of glucuronide conjugation from ion 699 and 654. Therefore, M13 was assumed to be a glucuronide conjugate of M5.

**12. Characteristics of M14**

At a retention time of 10.419 min, metabolite M14 (C_34_H_29_F_3_N_2_O_12_) was identified, and the molecular ion [M−H]^−^ was detected at *m/z* 713.1596, which was 176 Da larger than that of M6/M7. A characteristic ion at *m/z* 536.7586 was shown in Fig. S4(F), suggesting the loss of glucuronide conjugation. The breakage of CO_2_ at the E ring produced the fragment ion at m/z 492.7979. Those two ions above were the same as the product ions of M6/M7. Therefore, M14 was assumed to be a glucuronide conjugate of M6 or M7.
